# One Health: Gesundheit von Mensch, Tier und Umwelt

**DOI:** 10.1007/s00103-023-03712-5

**Published:** 2023-06-12

**Authors:** Suzan Fiack, Wolfgang Straff, Birgit Walther

**Affiliations:** 1 Bundesinstitut für Risikobewertung, Max-Dohrn-Str. 8–10, 10589 Berlin, Deutschland; 2 Umweltbundesamt, Corrensplatz 1, 14195 Berlin, Deutschland; 3 Zentrum für Biologische Gefahren und Spezielle Pathogene, Spezielle Licht- und Elektronenmikroskopie (ZBS4), Robert Koch-Institut, Seestr. 10, 14167 Berlin, Deutschland

„Man weiß, dass in Indien und in den Gegenden des großen Meeres die Gestirne … ihren Einfluss besonders auf jenes Meer ausüben und heftig gegen seine Gewässer ankämpfen. Daraus entstehen Dämpfe, welche die Sonne verdunkeln und ihr Licht in Finsternis verwandeln [1].“

So leitete im Jahr 1348 die medizinische Fakultät von Paris im Auftrag von Philipp VI. ein Gutachten zu den Ursachen der Pest ein. Die Prinzipien der Übertragung von Erregern von Infektionskrankheiten zwischen Mensch, Tier und Umwelt waren für die damaligen Gelehrten unvorstellbar. Erst Ende des 19. Jahrhunderts entdeckte Alexandre Yersin den Pesterreger, ein gramnegatives Bakterium, für dessen Verbreitung hauptsächlich der Rattenfloh ursächlich ist. Yersin brachte das Rattensterben in Hongkong in Zusammenhang mit den lokalen Pesttoten und schloss damit die entscheidende Erkenntnislücke bei der Infektkette zwischen Ratte, Bakterium, Floh und Mensch, was in der Folge eine effektive Bekämpfung der Krankheitsausbreitung ermöglichte.

Wie dieses Beispiel zeigt, sind die Gesunderhaltung und das Wohlergehen von Menschen, Tieren und der Umwelt eng miteinander verbunden. Viele Infektionserkrankungen, aber auch nichtinfektiöse Erkrankungen des Menschen stehen in einem engen bedingten Verhältnis zum Umweltzustand und der Gesundheit von Tieren. Die aktuellen und zukünftigen Herausforderungen und Probleme im Hinblick auf Gesundheitsfragen erfordern daher ein kollaboratives und interdisziplinäres Denken und Handeln – kurz einen One-Health-Ansatz. Das internationale Beratungsgremium „One Health High Level Expert Panel (OHHLEP)“ der WHO hat im Dezember 2021 eine operationelle Definition von One Health erarbeitet, um die gemeinsame Sprache für ein besseres Verständnis des One-Health-Ansatzes weiterzuentwickeln [2]. Im Zentrum steht dabei die interdisziplinäre und intersektorale Zusammenarbeit, um die Gesundheit von Menschen, Tieren und Ökosystemen nachhaltig ins Gleichgewicht zu bringen bzw. zu verbessern.

One Health hat in den vergangenen Jahren, z. B. aufgrund der COVID-19-Pandemie und des Auftretens der aviären Influenza, weiter an Bedeutung gewonnen, denn die Beziehungen zwischen Menschen, Tieren und Umwelt verändern sich laufend. Die menschliche Bevölkerung wächst und dringt zunehmend in unberührte Gebiete vor, dabei schaffen unter anderem der Klimawandel, die Veränderung der Landnutzung sowie die Veränderung von Lebensräumen für Menschen und Tiere neue Möglichkeiten für die Verbreitung von Krankheitserregern. Zudem sind Menschen, Tiere und Lebensmittel einschließlich tierischer Erzeugnisse durch Reiseverkehr und Handel international mehr in Bewegung als jemals zuvor.

Im Rahmen dieses Themenheftes werden aktuelle One-Health-Herausforderungen wie die Bedeutung bakterieller und viraler Zoonosen, die antimikrobielle Resistenz von Infektionserregern, die Lebens- und Futtermittelsicherheit sowie mögliche gesundheitliche Risiken durch die Bewässerung mit aufbereitetem Abwasser adressiert. Darüber hinaus werden neue Möglichkeiten bei der Bewältigung dieser Herausforderungen aufgezeigt, z. B. durch den Einsatz künstlicher Intelligenz. Eine weiterführende Perspektive setzt One Health in Beziehung zu dem verwandten Konzept „Planetary Health“ und stellt zusätzliche Themenfelder beider Ansätze vor, die über die im Rahmen dieses Heftes angesprochenen Aspekte hinausgehen.

Die Beiträge in diesem Heft zeigen, wie wichtig es ist, dem Thema Gesundheit mit einem breiteren Verständnis als bisher zu begegnen. Immerhin, zwischen dem Pestgutachten des 14. Jahrhunderts und dem One-Health-Ansatz des 21. Jahrhunderts ist schon viel Licht in die Finsternis gedrungen.

Eine Herausforderung bleibt die interdisziplinäre Betrachtung der umwelt- und gesundheitspolitischen Herausforderungen. Die Gesundheit der Menschen ist heute nicht weniger als in früheren Zeiten im Wesentlichen von den Lebensweisen, den Lebensbedingungen sowie dem Zustand der Umwelt abhängig. „Die Medizin ist eine soziale Wissenschaft, und die Politik ist weiter nichts als Medizin im Großen“, stellte schon Rudolph Virchow fest. Das Thema „One Health“ hat daher neben vielen fachlich interessanten Aspekten auch immer eine bedeutende politische Facette, die für die Gesundheit der Bevölkerung im Sinne von Public Health nicht unterschätzt werden sollte. Die Artikel werden deswegen durch eine Darlegung der Sichtweise thematisch involvierter Ministerien eingeleitet.

Unser Dank gilt Tomke Zschachlitz (UBA) und PD Dr. Bernd-Alois Tenhagen (BfR), die uns bei der Heftkonzeption unterstützt haben.

Wir wünschen Ihnen eine anregende Lektüre und verbleiben mit herzlichen Grüßen,

Suzan Fiack, Wolfgang Straff und Birgit Walther
